# Supplementary figures and images for: Lactoferrin-Derived Peptide Chimera Induces Caspase-Independent Cell Death in Multiple Myeloma
Source: Cells. 2025 Feb 3;14(3):217. doi: 10.3390/cells14030217 (PMC11817516; doi:10.3390/cells14030217)

Supplement figure S1

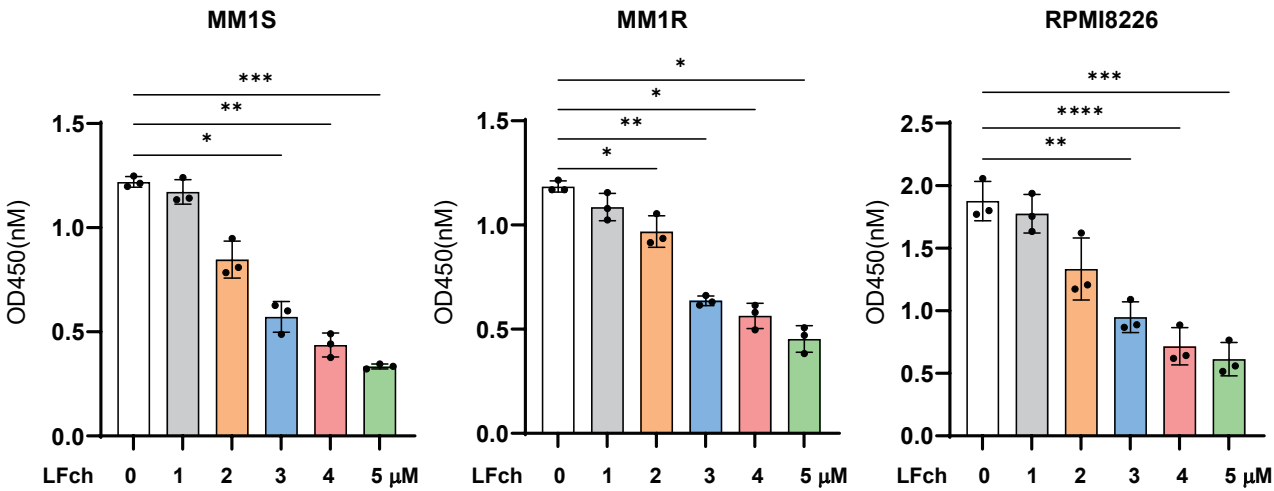

# Supplement figure S2

A

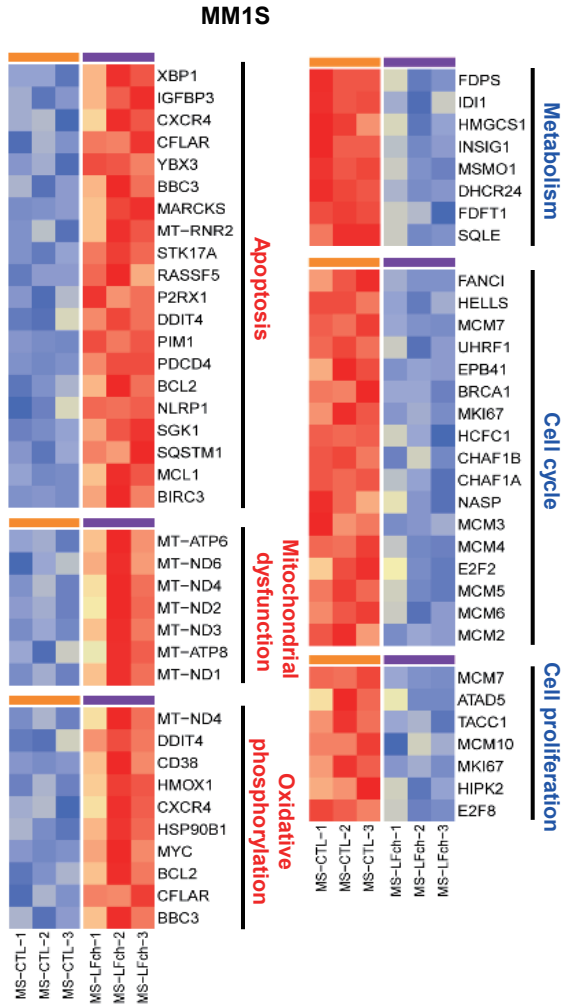

B

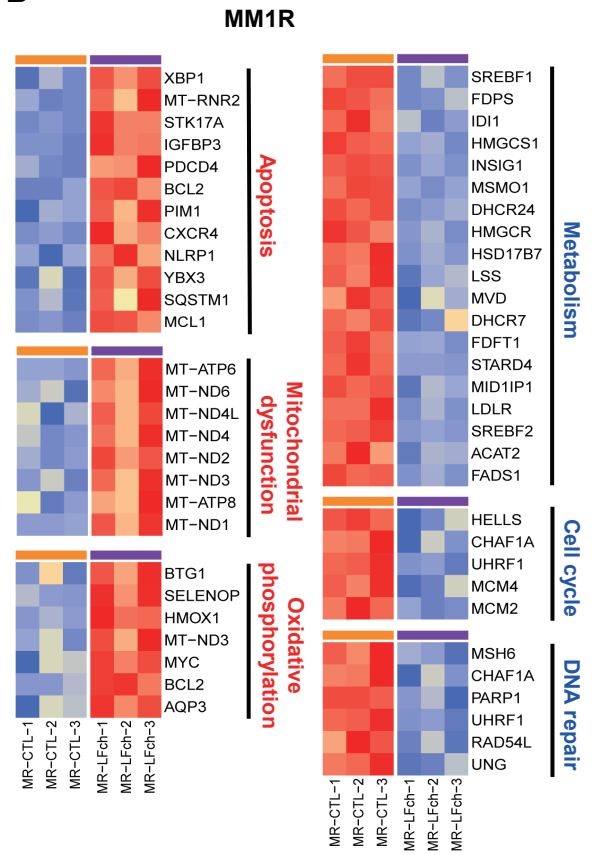

RPMI8226

C

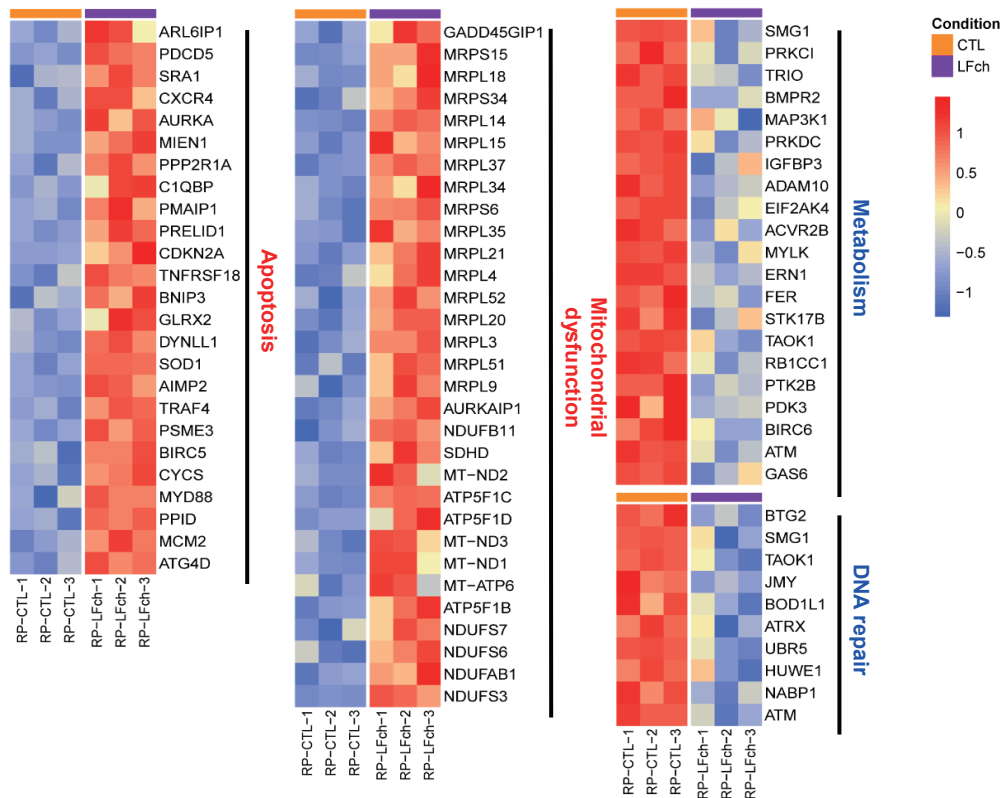

Supplement: Supplementary file 1 [file cells-14-00217-s001.zip › cells-3411006-supplementary.pdf]
